# A primer for quantum computing and its applications to healthcare and biomedical research

**DOI:** 10.1093/jamia/ocae149

**Published:** 2024-06-27

**Authors:** Thomas J S Durant, Elizabeth Knight, Brent Nelson, Sarah Dudgeon, Seung J Lee, Dominic Walliman, Hobart P Young, Lucila Ohno-Machado, Wade L Schulz

**Affiliations:** Department of Laboratory Medicine, Yale School of Medicine, New Haven, CT 06520, United States; Biomedical Informatics and Data Science, Yale School of Medicine, New Haven, CT 06510, United States; Yale School of Medicine, Yale University, New Haven, CT 06510, United States; Newport Healthcare, Minneapolis, MN 55435, United States; Department of Psychiatry, University of Minnesota, Minneapolis, MN 55454, United States; Computational Biology and Bioinformatics, Yale University, New Haven, CT 06510, United States; Department of Laboratory Medicine, Yale School of Medicine, New Haven, CT 06520, United States; Yale School of Medicine, Yale University, New Haven, CT 06510, United States; Domain of Science LTD, Bristol, BS37 6AG, United Kingdom; Department of Laboratory Medicine, Yale School of Medicine, New Haven, CT 06520, United States; Biomedical Informatics and Data Science, Yale School of Medicine, New Haven, CT 06510, United States; Department of Laboratory Medicine, Yale School of Medicine, New Haven, CT 06520, United States; Biomedical Informatics and Data Science, Yale School of Medicine, New Haven, CT 06510, United States

**Keywords:** quantum, quantum computing, biomedical research, healthcare, quantum annealing, universal gate-based quantum computing

## Abstract

**Objectives:**

To introduce quantum computing technologies as a tool for biomedical research and highlight future applications within healthcare, focusing on its capabilities, benefits, and limitations.

**Target Audience:**

Investigators seeking to explore quantum computing and create quantum-based applications for healthcare and biomedical research.

**Scope:**

Quantum computing requires specialized hardware, known as quantum processing units, that use quantum bits (qubits) instead of classical bits to perform computations. This article will cover (1) proposed applications where quantum computing offers advantages to classical computing in biomedicine; (2) an introduction to how quantum computers operate, tailored for biomedical researchers; (3) recent progress that has expanded access to quantum computing; and (4) challenges, opportunities, and proposed solutions to integrate quantum computing in biomedical applications.

## Introduction

Quantum computing is an emerging field with the potential to accelerate computation in many areas of science and technology.^1^^,^^2^ Based on the principles of quantum mechanics, quantum computing uses a fundamentally different approach than classical computing.^3^ Because of this, quantum computers (QCs) have the potential to perform complex calculations more efficiently than classical computers, and thus may offer promising applications within healthcare and biomedical research.^4^^,^^5^

Quantum computing has several possible benefits over classical computing in biomedical problems that have high computational complexity (Table 1).^6^ For example, QCs can be used to optimize solutions for complex problems that have an inherently underlying quantum structure, such as simulating molecular interactions and protein folding.^5^^,^^7^^,^^8^ Similarly, quantum algorithms, such as Grover’s algorithm, can search large, unstructured datasets more efficiently than their classical counterparts, enabling more effective optimization of complex problems in healthcare and biomedical research.^4^^,^^9^ However, there are also notable drawbacks, including current limitations in error correction, hardware scalability, and the need for specialized programming skills to effectively use quantum computing resources.^10–12^

**Table 1.** Areas of application in healthcare and biomedical research.

Area of application	Description	References
Artificial intelligence and machine learning	Quantum computing brings novel algorithms for machine learning applications, with recent publications demonstrating potential advantages for feature selection and classification models.	^10^ ^,^ ^26–32^ ^,^ ^34–40^ ^,^ ^101^ ^,^ ^102^
Genomics	Quantum computing may help with the analysis of large-scale genomic data, leading to improved understanding of genetic diseases and personalized medicine. Quantum algorithms like Grover’s search algorithm and its variations may be employed to search and analyze vast genomic databases more efficiently than classical methods.	^25^ ^,^ ^41–47^ ^,^ ^103^
Protein folding	Understanding protein folding is essential for the study of diseases related to protein misfolding, such as Alzheimer’s and Parkinson’s. Quantum computing can potentially improve the efficiency of predicting protein folding patterns. Algorithms like the Quantum Approximate Optimization Algorithm (QAOA) can be used to tackle optimization problems, including protein folding.	^48–52^
Drug discovery	Quantum computing could significantly accelerate the drug discovery process by simulating molecular interactions and predicting the behavior of drug candidates. Quantum algorithms like the Variational Quantum Eigensolver (VQE) and Quantum Phase Estimation Algorithms (QPEA) can be used to determine molecular ground-state energies, which is crucial for understanding molecular properties and reactions.	^5^ ^,^ ^53–61^
Network analysis	Network analysis in systems biology, utilized extensively in bioinformatics, involves tasks like molecular modeling and mapping complex biological pathways. Quantum computing has been applied to enhance community detection within networks, offering approaches that are comparable to classical methods. This has been described using quantum annealing and other quantum-based algorithms, which show promise in effectively partitioning networks into communities, a critical step in understanding biological and computational systems more deeply.	^62–66^
Cryptography	Quantum computing poses a significant challenge to current encryption methods like RSA, as quantum algorithms, such as Shor’s algorithm, can efficiently factor large prime numbers, potentially undermining these encryption schemes^58^. Conversely, it also enables the development of quantum-resistant encryption techniques, such as Quantum Key Distribution (QKD), which may enhance modern cybersecurity.	^67^ ^,^ ^68^ ^,^ ^70^ ^,^ ^73^

The accessibility of quantum resources has rapidly expanded with the general availability of commercially available hardware, high-level programming languages, and cloud-hosted resources, including central processing unit (CPU)-based simulators and quantum processing units (QPUs).^13^ Despite this progress, the practical application of quantum computing to specific tasks remains a challenge, with limited examples available for practitioners who may not have extensive experience in low-level programming or quantum concepts.^11^

By harnessing the power of quantum computing, certain problems that were previously intractable or extremely time-consuming may lead to new insights and discoveries. However, this may also work against many applications in healthcare, particularly those that rely heavily on encryption as a primary means to protect data. In this manuscript, we present an overview of quantum computing principles, specifically tailored to biomedical researchers. We provide examples of how and when quantum computing can be applied to biomedical and healthcare applications. Lastly, we provide a high-level overview of recent progress that has expanded access to quantum computing, as well as the challenges, opportunities, and proposed solutions to integrate quantum solutions into biomedical computing.

## Impact of quantum computing on healthcare and biomedical research

**Figure 1.**
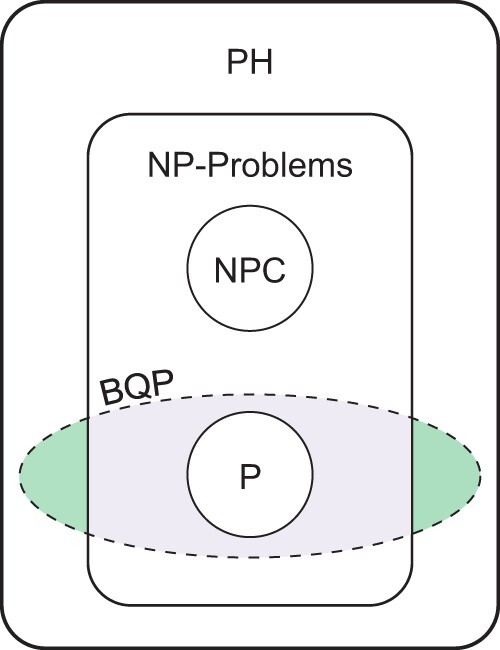
Computational complexity theory has traditionally included 3 major complexity classes: polynomial-time (P), non-deterministic polynomial-time (NP), and NP-complete (NPC), all of which are contained in polynomial hierarchy (PH) space. Bounded-error quantum polynomial time (BQP) is a complexity class that represents problems that should be efficiently solved by a quantum computer. Problems that fall within this space and the bounds of the BQP space itself remain an open question, with recent literature indicating that it may not be constrained by the PH-space. Depending on the bounds of the BQP-space, there may be problems that will only be efficiently solved by quantum algorithms. Figure adapted from Nielsen and Chuang^6^.

The strength of quantum computing resides in its capacity to simultaneously represent multiple possibilities through quantum parallelism, which stems from the quantum properties of superposition, entanglement, and interference, described further in the following section.^6^ Computational complexity theory serves as a framework for understanding the boundaries and conditions in which quantum computing resources can demonstrate a proven advantage (Figure 1).^14^ However, it remains an open and crucial question as to whether QCs can more efficiently solve problems that are intractable for classical computers.^15^ Classical computational complexity theory revolves around the P (polynomial time) and NP (nondeterministic polynomial time) classes. Problems in P can be solved quickly (in polynomial time) by classical computers, whereas NP problems have solutions that can be verified quickly but may not be solvable quickly. QCs introduce new complexity classes, like BQP (bounded-error quantum polynomial time).^14^ This class is significant because some problems that are believed to be outside of P (and potentially in NP) for classical computers, like integer factorization, fall into BQP, suggesting that QCs may be able to solve them efficiently.^14^ These problems are of practical note since efficient quantum solutions may lead to new efficiencies in genomic data analysis, molecular simulation, or encryption technology (or, potentially, breaking current encryption algorithms).^16^^,^^17^ Quantum algorithms have also shown promise in machine learning (ML); however, while some problems in ML can be analyzed in terms of computational complexity theory, ML as a whole is not defined by these classes.

Despite our limited understanding of how and when to use QCs to tackle real world problems, recent literature has only just started to identify near-term areas where quantum computing applications may achieve quantum advantage.^4^ In the domain of biomedicine, there exists a wide range of computationally intractable problems that intuitively lend themselves to quantum computing, with algorithms that appear to be well-suited for these challenges (Table 1). As quantum computing resources continue to mature, applied research in this domain is expected to grow. We anticipate that, mirroring the trends in ML, inter-disciplinary collaboration will speed the development and use of quantum algorithms.

## How quantum computing works

### Quantum advantage

Superposition and entanglement are widely recognized as critical features in quantum computing, which may afford it significant advantages over classical computing.^6^ In addition, several other quantum phenomena, such as coherence, contextuality, interference, and superdense coding, may also play pivotal roles.^18^ For the interested reader, further information on additional quantum mechanical properties and how they may be leveraged to accelerate computing can be found in recent literature.^18^^,^^19^ In addition, it is important to note that the following sections will focus on principles that are primarily employed in gate-based QCs.

### Bits and qubits

**Figure 2.**
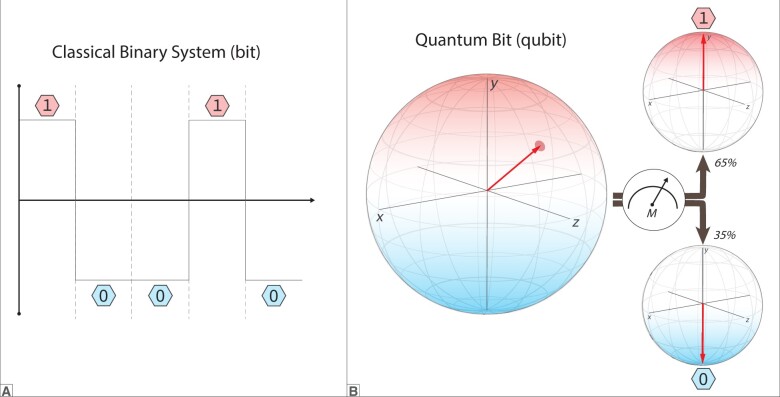
(A) Classical bits, the fundamental units of information in classical computing, represent binary data as either a 0 or a 1, typically by utilizing voltage levels within an electronic system. By flipping the voltage between 2 predefined levels, such as a high voltage to signify a 1 and a low voltage for a 0, the state of a bit can be easily switched, stored, and transmitted. The use of voltage levels in this manner allows for a straightforward, reliable method of encoding and processing digital information. (B) Quantum bits, or qubits, are the fundamental units of quantum computing, and unlike classical bits, they can exist in a superposition of both 0 and 1 states simultaneously. This unique property is made possible by the principles of quantum mechanics, particularly wave-particle duality and superposition. The actual state of a qubit can be described by a complex probability amplitude that defines the likelihood of observing either a 0 or a 1 upon measurement. When a qubit is measured, its superposition collapses to one of the two classical states (0 or 1).

In classical computing, a bit is the fundamental unit of information, represented by either a 0 or a 1 (Figure 2A).^20^ These bits are used by traditional computing hardware to store data and are manipulated through a series of mathematical and logical operations to perform computational tasks. The most notable feature of bits in this context is that bits can only be in 1 of 2 states (0 or 1), and each bit exists in a state that is independent from others such that the state of one bit does not directly influence the state of another bit.^6^ In contrast, a qubit, or quantum bit, is the fundamental unit of information used in QCs (Figure 2B). Qubits are minimalistic, physical systems where the 0 and 1 states are encoded onto a 2-level quantum state that displays the unique properties of quantum mechanics, including superposition, entanglement, and interference.^1^

### Superposition and measurement

Superposition refers to the ability of a quantum object to simultaneously exist in a mixture of states.^20^ In contrast to classical bits, this means that qubits can exist in a continuum of states between 0 and 1. However, if a measurement is performed, the probabilistic nature of states will cease to exist, and the state of the qubit will resolve to a base state of 0 or 1 (Figure 2B).^6^ In the context of quantum computing, measurement refers to the process of extracting classical information from a quantum system, such as a qubit, by observing its state. Measurement plays a crucial role in quantum computing, as it bridges the gap between the quantum realm and the classical world.^6^^,^^20^ Importantly, the superposition state of a qubit is unobservable, but the superposition state can be manipulated prior to measurement which results in experimentally observed differences in the probability of outcomes.^6^^,^^20^

### Entanglement

The study of quantum systems has shown that, under specific conditions, when 2 particles are prepared in close physical proximity to each other, the superposition state of particle A and particle B will strongly correlate with each other, even after the particles are physically separated over large distances, which is known as entanglement.^2^ Experts in quantum computing have shown that entanglement can be leveraged as a key feature that may allow QCs to gain a computational advantage over classical hardware for certain computational problems.^20^

In quantum computing, a system of *n* qubits can be prepared in an entangled state such that their individual states are no longer mathematically separable. When the 2 qubits are measured, the possible results 00, 01, 10, or 11 will be observed according to a probability distribution defined by the entangled quantum state.^1^^,^^6^^,^^20^ This represents a novel approach to computing, since the individual state of a classical bit does not directly influence other bits. If we consider a system of *n* qubits, the quantum state is defined by 2^*n*^ amplitudes.^1^^,^^20^ In practice, the number of possible states in a system of 300 qubits would be larger than the number of atoms in the observable universe.^6^ While a similar principle applies to classical computers, quantum computing can exploit this characteristic without the need for the user to concurrently manage all state permutations, which constitutes a distinct advantage over classical computers.^1^^,^^20^

### Quantum computation and matrix representation

Mathematically, qubits and their associated quantum properties can be described using linear algebra. Accordingly, the 2-level superposition state of a qubit can be described mathematically as the following:

|ψ⟩=α|0⟩+β|1⟩. 

The | and ⟩ is called *Dirac notation* and it is used as a reminder that the enclosed variable is a vector.^6^^,^^20^^,^^21^ In this case, the following qubit states can be written as:

|0⟩=10 and |1⟩=01.

Here, *α* and *β* are complex numbers and are referred to as *amplitudes.* The square of these amplitudes represents the probability of a qubit being in either of the corresponding states. The exact values of *α* and *β* cannot be precisely determined because, when a qubit is measured, the superposition state of the qubit will collapse to either 0 with a probability of ${\left|\alpha \right|}^{2}$ or 1 with a probability of ${\left|\beta \right|}^{2}$. The probabilities sum to 1 and therefore ${\left|\alpha \right|}^{2}+\;{\left|\beta \right|}^{2}\;=\;1$.

### Universal gate-based QCs

**Figure 3.**
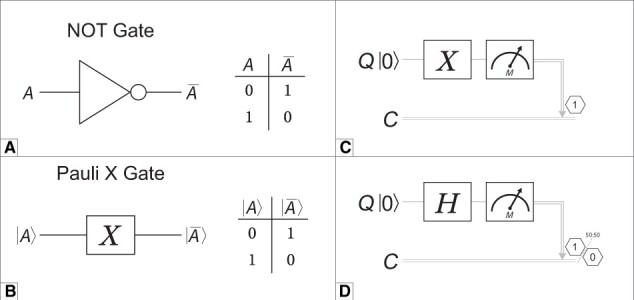
(A) The classical NOT gate, which inverts the input (A) to produce the output (Ā) indicated in the adjacent truth table indicating classical register output. (B) The quantum Pauli X gate, which operates on a quantum bit (qubit) in state |A⟩ and yields the inverted state |Ā⟩, analogous to the classical NOT operation. Both gates demonstrate the inversion principle where an input of 0 yields a classical register output of 1, and vice versa. (C) Quantum circuit diagram illustrating the initialization, manipulation, and measurement of a single qubit. The qubit Q starts in the ground state |0⟩, followed by the application of a Pauli-X gate (X) which inverts the state to |1⟩. A measurement operation (M) is then performed, the result of which is recorded in the classical register C. The output is indicated as “1,” representing the classical bit obtained post-measurement. (D) Quantum circuit diagram with the Hadamard (H) gate, which encodes the input data into quantum superposition, resulting in a probability of results post-measurement based on the inputs and circuit composition.

Classical computers operate on traditional bits through operations called logic gates, such as AND, XOR, and NOT.^22^ Logic gates are a hardware abstraction layer that represent the fundamental logic upon which classical computer algorithms are built.^22^ QCs can offer a similar layer of abstraction to leverage quantum states as a general-purpose register (small storage areas inside a processing unit that temporarily hold data for rapid access during processing).^20^ Computations on quantum states are facilitated by linear operators referred to as quantum logic gates.^6^ QCs that follow this paradigm are known as universal gate-based QCs.^23^ Classical and quantum logic gates are similar in that both take a set of inputs and produce an output. Some logic gates are analogous between classical and quantum systems. For example, the classical NOT gate (Figure 3A) and quantum Pauli X gate (Figure 3B) and their corresponding truth tables demonstrate that the logical output of these gates is equivalent.^6^

While quantum and classical computing gates are conceptually analogous, there are also key differences. A significant difference is the absence of conventional data flow through a quantum gate. As quantum gates are applied to a register of qubits, the state probabilities are changed, influencing the eventual outcomes when these qubits are measured.^20^ The measurement process translates the quantum information into a classical form, which is read out to a classical register, providing results that can be used as a computing resource.^6^ Another key difference in quantum computing is that quantum gates are reversible, meaning that for a given output there is a known input.^6^ This follows a fundamental theorem in quantum mechanics that information cannot be lost or destroyed. This constraint is not true for all classical logic gates, such as the AND gate.

Despite these differences, both classical and quantum logic gates are similarly combined into circuits. For the interested reader, Nielsen and Chuang provides a comprehensive review of the details associated with these quantum operations.^6^ Quantum circuits can take complex forms, but a simple circuit can demonstrate a computation analogous to a classical bit-flip (Figure 3C, D). Quantum circuits are read from left-to-right, with the top line representing a quantum register containing information associated with one qubit (Q). The bottom-most line (C) represents a classical register consisting of 1 bit for storing the qubit measurement (M). In this circuit, qubit Q is initialized in a base state of |0⟩, flipped to a state of |1⟩, and measured (M). The output of this quantum circuit would yield a state of 1 on the classical register 100% of the time (Figure 3C). If, after initialized, the Hadamard (H) gate is used to encode the data into quantum superposition, the classical register from the circuit would yield a state of 0 approximately 50% of the time and 1 approximately 50% of the time (Figure 3D).

Similar to how qubits are represented in vector form, quantum gates and circuits can also be represented with linear algebraic matrices. The X-gate (ie, Pauli X Gate) can be written as:

Pauli X≡0 11 0.

Taken together with the qubit-vector representation, the circuit described in Figure 3C would be written as:

|0⟩= 0 11 010= 01= 1⟩.

More detailed examples of the mathematical representation of quantum gates and circuits can be found in several references and example circuits with their resulting output simulated with software tools.^6^^,^^24^

## Applications of quantum computing in healthcare and biomedical research

Recent publications have increasingly described the application of QCs in specialized areas of biomedical informatics, such as ML, protein folding, and drug discovery.^12^^,^^17^ This section offers a practical overview of promising research avenues, open questions, and quantum-based computational methods in biomedical informatics. However, it is important to note, recent applications of quantum computing bioinformatics are constrained by scale, as current QCs are limited in size and error correction capabilities, preventing them from handling full-sized datasets.^25^ Therefore, developing larger and more fault-tolerant QCs remains essential for achieving practical quantum advantage in these fields.

### Artificial intelligence and machine learning

Quantum computing offers potential benefits in predictive performance and computational scalability for artificial intelligence (AI) and ML applications, particularly for tasks such as classification, regression, clustering, and feature selection.^10^^,^^26^^,^^27^ However, because classical ML algorithms cannot be directly implemented on quantum hardware, the development of quantum machine learning (QML) algorithms has become an area of focus in this emerging field.^10^ Over the past decade, quantum adaptations of classical algorithms have been described, such as quantum support vector machines (QSVMs),^28^ quantum neural networks (QNNs),^29^ variational quantum classifiers (VQCs),^30^ and quantum kernel estimators,^30^ along with other QC-based algorithms without clear classical analogs.^31^

Recent applications of QML in biomedical and healthcare research include evaluating brain MRI images,^32^ predicting patient responses to cancer drugs,^33^ and detecting the presence or absence of diseases.^34–36^ In studies that have compared classical ML and QML, the performance of QML models are often similar or underperform, relative to classical counterparts.^32^^,^^34^ It is suggested that future comparative studies in this area may help identify datasets that better leverage QML algorithms and identify optimization opportunities for QML algorithms.^34^^,^^37^

There has also been research into reducing model complexity and improving interpretability through quantum-based feature selection.^32^^,^^38^^,^^39^ This work predominantly employs quantum annealing (QA) for feature selection. For this approach, correlations such as mutual information or Pearson correlation matrices are converted into quadratic unconstrained binary optimization (QUBO) problems, which represent the total energy of the system.^40^ QA can then be leveraged to minimize this energy, where the optimal features correspond to the system’s ground state.^32^^,^^38^^,^^39^ But much like QML, the benefits of quantum feature selection in terms of performance, computational scalability, and feature stability are yet to be fully established.^32^

### Genomics

As the use of high-throughput sequencing continues to increase, so does the need for robust and scalable data analytics solutions for genomic data.^41^ Given the raw size and complexity of genomic data, it is an ideal candidate for exploring QC-based solutions which may offer better accuracy and performance across many bioinformatics tasks.^17^ To this end, recent publications have demonstrated the successful implementation of quantum-based algorithms for *de novo* and reference-based DNA sequence assembly.^41–44^ Others have also implemented QC-based solutions for variant detection,^25^ reconstruction of phylogenetic trees,^45^^,^^46^ and the detection of epistatic interactions between genomic single nucleotide polymorphisms.^47^ In these studies, the authors demonstrate that QC-based methods are feasible and accurate but note the limited ability to scale due to the limited size and lack of error correction in current QCs.^41^

### Protein folding

Predicting 3-dimensional structures of proteins is a widely researched problem. Recent studies that have employed QC-based algorithms typically frame this challenge as an optimization problem. The objective of this problem is to identify the ground state energy representing the most stable protein conformation based on a model of the protein’s interactions and conformations.^17^^,^^48^^,^^49^ Numerous publications have evaluated this approach and investigated the use of adiabatic algorithms, such as QA, and circuit-based algorithms, the latter of which often use variational quantum algorithms (VQAs).^48–50^ Collectively, these works have demonstrated that subsampled protein folding problems can be successfully integrated and executed on modern QCs. These results are encouraging as they suggest potential speed enhancements over traditional optimization algorithms and are expected to motivate further research into scaling these methods to larger protein models as advancements in quantum computing technology occur.^17^^,^^48^^,^^51^^,^^52^

### Drug discovery

Computer-aided drug discovery (CADD) plays an integral role in the drug discovery and development process, encompassing tasks such as predicting drug-target interactions, performing energy calculations, and conducting virtual screenings.^53^^,^^54^ Given the fundamental properties of quantum mechanics, QC-based methods are seen as a promising avenue of investigation for enhancing currently available CADD approaches.^55^ A number of CADD tasks have recently been implemented on quantum-based hardware and simulators.^12^ Lau et al investigated the use of QNNs for predicting mutational effects on drug binding properties, but showed equivocal performance compared to classical neural networks.^5^ In addition, variational quantum eigensolvers (VQEs) were used to compute protein-ligand activation energies; however, this was done using QC-simulators under noiseless conditions.^56^ Similarly, Kirsopp et al, also employed VQEs for calculating protein-ligand binding energies and successfully implemented their approach on IBM and Quantiniuum QC hardware, demonstrating comparable results with classical approaches.^57^ Additional studies have examined the use of quantum computing for predicting molecular docking configurations,^58^^,^^59^ QNNs for predicting molecular force fields,^60^ and hybrid quantum generative adversarial networks (qGANs) and quantum variational autoencoders (qVAEs) to generate small and large drug molecules, respectively.^61^ As with many of the other recent applications of quantum computing, the work in this area remains exploratory and is often implemented in simplified systems to highlight prototypical use cases that work on available QC hardware.^57^

### Network analysis

Network analysis, a common framework used in systems biology, is extensively leveraged in bioinformatics for purposes such as molecular modeling, mapping intracellular biological pathways, and representing neuronal pathways.^62–64^ Detecting relationships among the constituents of complex systems has evolved into a dedicated field of study, known as community detection.^65^ This area focuses on the accurate and efficient partitioning of networks into communities, leading to the development of graph partitioning algorithms to address this NP-hard problem.^63^^,^^65^ To date, several publications have shown proof-of-concept implementations of quantum-based algorithms for community detection. Recently, Negre et al, described the implementation of QA for community detection across various benchmark network datasets, demonstrating that the quantum-based method was comparable to classical methods.^63^ In biological applications, Wierzbiński et al explored the use of D-Wave’s quantum annealers for community detection within brain connectomes. They compared this approach to the Louvain Community Detection Algorithm and demonstrated superior performance with QA when using higher modularity as a proxy for cluster quality.^66^

### Cryptography

In the United States, protected health information (PHI) is defined and regulated primarily by the Health Insurance Portability and Accountability Act of 1996 (HIPAA) and the Security Rule of 2003. These regulations mandate that PHI be handled with care and confidentiality, often necessitating the use of cryptography. Accordingly, the development of quantum algorithms such as Shor’s and Grover’s, which can solve mathematical problems foundational to many modern public key cryptography algorithms, carries significant implications for the security of healthcare data.^12^ Recent publications have explored the use of both gate-based QCs and QA for integer factorization, which is a core mathematical principle of Rivest–Shamir–Adleman (RSA)-based cryptographic methods.^67–69^ However, currently available QCs do not yet pose a meaningful threat to modern cryptography, and cryptanalytically relevant QCs are unlikely to become available before 2030.^70^ Nonetheless, in response to these potential threats, governmental agencies have begun to develop and release 4 quantum-resistant cryptographic algorithms, with mandates to migrate federal systems to post-quantum cryptographic methods by 2035.^71^^,^^72^ Despite these proactive steps, there is a theoretical risk that data, if encrypted and stored today, could later be decrypted by more advanced, fault-tolerant QCs in future “harvest-decrypt attacks”.^73^

## Quantum hardware and software: current state

The field of quantum computing has undergone remarkable advancement over the last decade, most notably evidenced by the development of universal gate-based QCs by multiple vendors including Microsoft, Honeywell, IBM, Google, and IonQ.^74–77^ In recent years, these systems have been made accessible via cloud-hosted platforms, which has enabled developers to deploy quantum algorithms on a diverse assortment of quantum hardware. The provision of cloud-based quantum computing resources presents a novel avenue for exploration within biomedical research. Given the accelerated evolution of quantum computing, it will be essential for researchers in their respective domains to identify potential applications and assess where it confers an advantage over classical computing. Accordingly, this section provides a high-level overview of the hardware and software development kits (SDKs) currently available to the research community to facilitate the acquisition of practical, hands-on experience with QCs.

In terms of quantum hardware, the current landscape of quantum computing can be divided into several paradigms which sometimes overlap in their implementation: gate-based quantum computing, QA, adiabatic quantum computing, quantum simulation (with both gate-based and non-gate-based variants), topological quantum computing, and measurement-based quantum computing.^78^ Gate-based quantum computing implements operations via quantum gates, drawing parallels to how classical computing operates with bits.^6^ QA and adiabatic quantum computing direct quantum systems to evolve naturally under specified energy configurations, circumventing the necessity for gates.^79^ Quantum simulation uses one quantum system to mimic another and can be implemented with either gate-based or non-gate-based approaches.^19^ Topological quantum computing leverages particular quasi-particles and their movement patterns, providing inherent resistance to errors owing to their unique properties.^80^ Conversely, measurement-based quantum computing conducts computations by performing a sequence of measurements on a pre-prepared, highly entangled state, also known as a cluster state.^81^ Despite the diversity of these paradigms, the close alignment of gate-based systems with classical computing principles has led to this being the predominant method employed and explored by many quantum computing companies and algorithm developers.^74–76^

The availability of quantum computing resources varies across vendors, but universal gate-based QPUs are most common. At the time of this writing, these QPUs typically possess between 50 and 100 qubits, with some implementations offering up to 400+ qubits (IBM Osprey).^76^ However, access for most non-paid accounts is usually confined to QPUs with fewer qubits, for instance 5-10. While paid accounts are available, their cost is prohibitive for many academic endeavors. In response to this, cloud providers extend a variety of academic subscriptions that allow researchers to secure dedicated time on smaller QPUs, in addition to enabling quantum computer simulations on classical computers. These simulators widen the accessibility of quantum development as they typically have shorter queue times and are generally offered free of charge. Quantum simulators can be employed to test and optimize algorithms iteratively using a selected quantum software development kits (QSDKs) prior to submitting jobs to actual quantum hardware, considering that queue times for these shared and limited resources vary.

Some vendors also provide access to non-gate-based QCs, such as the QA platform offered by D-Wave (Advantage/LEAP).^82^ Diverging from gate-based QCs, the QA systems do not typically rely on circuits or logic gates and are tailored towards resolving optimization problems.^83^ Accordingly, problems submitted to these quantum systems are represented as a specific mathematical function, such as a QUBO problem, that can subsequently be mapped onto the QPU. This mathematical function is then optimized through iterative sampling of the quantum system toward a target energy state. QA forms part of a subset of a larger quantum computing paradigm known as adiabatic quantum computing.^84^

**Figure 4.**
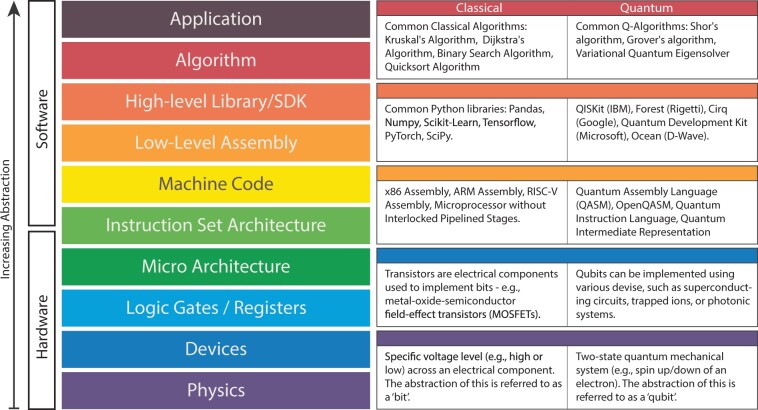
Hierarchical layers of abstraction in computer systems, both classical and quantum. Each layer represents a step up from the physical components that execute basic operations to the sophisticated applications that interact with users. It illustrates how quantum computing parallels and diverges from classical systems, providing a visual comparison of their distinct computing models at each abstraction level. Figure adapted from Fu et al.^89^

To interact with quantum hardware and build quantum applications, QSDK and other layers of abstraction have been developed (Figure 4). These SDKs offer abstractions and tools for interfacing with quantum hardware, enabling developers to manipulate quantum algorithms, gates, and circuits in higher level languages, such as Python and .NET. Examples of QSDKs include IBM’s Qiskit, Microsoft’s Quantum Development Kit (QDK), and Google’s Cirq.^74^^,^^85–88^ While the physical realization of qubits varies among vendors (eg, superconductor and trapped ion), the application of quantum logic gates remains consistent, facilitating a universal approach to interact with the various gate-based systems. To this end, QSDKs are designed to work both with specific quantum hardware and across platforms; however, the authors have observed that cross-platform compatibility and performance is not universally consistent. There is hope that, as QSDKs and specialized quantum libraries for methods such as QML mature, this interoperability will continue to improve.

Overall, the current landscape of quantum computing is characterized by a diverse array of quantum hardware and QSDKs. This diversity of resources, marked by differences in design and computational paradigms, offers unique capabilities and constraints that researchers can leverage. QA-based QCs, for example, can possess a substantial number of qubits, reaching up to 5000. However, not all computational problems are readily adaptable to the format required by these non-gate-based systems. Gate-based QCs, conversely, are seen as offering a more adaptable computing framework that can function independently or in conjunction with an analog-like structure. Nonetheless, these gate-based systems grapple with issues related to scalability and are currently limited in the number of qubits they can support in comparison to their non-gate-based counterparts. Ultimately, the choice between gate-based and non-gate-based QCs hinges on the specific requirements of the algorithm or task in question, making the variety of available quantum computing resources a substantial asset to the field. The continued evolution of these systems and development of their accompanying software tools promise to further expand the possibilities of quantum computing research and applications.

## Integrating quantum computing with today’s data science platforms

Historically, CPUs fulfilled the role of executing general-purpose computing tasks, including those related to ML. However, as ML models evolved in complexity and consequently demanded increased computational capacity, the limitations of relying solely on CPUs became apparent. In the mid-2000s, ML researchers adapted graphics processing units (GPUs) to substantially expedite the training process of deep neural networks, which had been a considerable impediment in the advancement of ML models.^90^ In response to this, manufacturers released specialized GPUs and tensor processing units (TPUs) tailored for ML applications.^91^ These innovations provided more cost-efficient solutions for ML tasks, enabling a greater capacity for model complexity and performance.

Just as the accessibility of GPUs for ML empowered organizations to train more complex models, the availability of QPUs will likely foster the development of more advanced quantum algorithms, thereby catalyzing new applications and use cases. Since not every computation or algorithm will achieve higher speed or performance on quantum hardware, QPUs should be viewed as an accelerator to CPU-based computing and considered as complementary hardware, rather than as a replacement for conventional computing systems. Therefore, incorporating this emergent technology into increasingly modular system architectures will enable developers to select the most suitable hardware based on the computational tasks being devised.

## Challenges and opportunities

One of the primary impediments to the progress of quantum computing lies in achieving and maintaining quantum coherence, or the quantum state, of qubits for extended periods.^92^ Physical qubits exist in a delicate state and are sensitive to environmental interference, which can introduce noise into the system.^6^ Factors like imperfections in the physical qubit, temperature fluctuations, and electromagnetic interference can disrupt their state, leading to a phenomenon known as decoherence.^6^^,^^92^ Efficient error correction mechanisms are vital to the functionality of QCs. However, current error correction methods are resource-intensive, wherein some approaches require a significant number of physical qubits to protect a single logical qubit. Finally, building scalable quantum systems that can effectively leverage quantum entanglement and superposition to perform complex computations remains a substantial technical hurdle, limiting the number of qubits available in individual systems.

Given these challenges, there is a need for robust evaluation methods and benchmarking tools to assess quantum computing performance.^93^ The DiVincenzo criteria act as foundational guidelines, outlining the essential requirements for a practical and reliable quantum computer.^94^ These criteria emphasize the importance of coherence, scalability, error rates, and the functionality of quantum gates, among other aspects. Related to these criteria, benchmark metrics such as quantum volume offer a comprehensive approach to provide a unified measure of a device’s overall capability.^95^ Such metrics can be used to compare quantum computing systems by normalizing the variations due to system design and functional heterogeneities.

In addition to the current limitations of quantum hardware, direct applications of quantum-based algorithms to real-world problems remain limited. Notable quantum algorithms, such as Shor’s and Grover’s algorithms, demonstrate theoretical benefits over their classical counterparts by addressing well-defined tasks like factorization and unstructured search.^9^^,^^96^ Recent studies have shown proof of concept applications that have theoretically achieved quantum advantage over classical computing approaches.^19^^,^^97^^,^^98^ However, bridging the gap between theory and real-world use remains challenging.

Lastly, quantum computing is an inherently multidisciplinary field that necessitates collaboration among experts from computer science, mathematics, physics, and target application areas, including biomedical research and healthcare. The proliferation of cloud based QPUs and the provision of associated development environments hold the potential to foster collaborative efforts and information sharing across these diverse fields. However, widespread adoption of quantum computing hinges on numerous contributing factors, including the maturation of dedicated software tools, a deeper understanding of where and how quantum advantage can be achieved, and the establishment of easily accessible quantum computing infrastructures integrated with existing platforms.

## Conclusion

Quantum computing holds significant promise in addressing certain challenges encountered by classical computing, particularly concerning complex analyses and optimization.^99^ Traditionally, biomedical researchers have grappled with scaling complex algorithms, such as those simulating chemical reactions or biological structures. The computational complexity of these analyses often overwhelms classical computing. The sheer number of variables involved in these processes leads to an exponential increase in the complexity of comprehensive and accurate models.^4^^,^^100^ Classical simulation then becomes computationally intractable, often necessitating the adoption of simplified models and/or approximations. In these scenarios, quantum computing offers a theoretical advantage in that it can potentially solve certain problems that are computationally intractable for classical computers.^5^

Despite the theoretical advantages of quantum computing compared to its classical counterparts, tangible demonstration of these benefits for real-world, previously intractable problems remains limited. Nevertheless, the emergence of quantum computing resources presents an opportunity to bolster collaborative endeavors across varied domains of expertise. By emulating the interdisciplinary approach adopted within AI and ML, there exists the potential to broaden and deepen the application of quantum technology. In parallel to the ongoing technological advancements, exploratory initiatives within quantum algorithm and application development may precipitate significant breakthroughs in biomedical research and healthcare, potentially effecting profound changes in these fields.

## Data Availability

No new data were generated or analyzed in support of this research.
